# The mediating role of job satisfaction in the effect of green transformational leadership on intention to leave the job

**DOI:** 10.3389/fpsyg.2025.1490203

**Published:** 2025-02-17

**Authors:** Onur Oktaysoy, Ethem Topcuoglu, Asli Ozge Ozgen-Cigdemli, Erdogan Kaygin, Gozde Kosa, Burcu Turan-Torun, Mehmet Selman Kobanoglu, Selen Uygungil-Erdogan

**Affiliations:** ^1^Faculty of Economics and Administrative Sciences, Kafkas University, Kars, Türkiye; ^2^Academy of Civil Aviation, Giresun University, Giresun, Türkiye; ^3^Kadirli Faculty of Applied Sciences, Osmaniye Korkut Ata University, Osmaniye, Türkiye; ^4^Faculty of Tourism, Van Yuzuncu Yil University, Van, Türkiye; ^5^Faculty of Political Sciences, Samsun University, Samsun, Türkiye

**Keywords:** green transformational, leadership, intention to leave the job, job satisfaction, organizational behavior

## Abstract

**Introduction:**

The literature on leadership has evolved in accordance with changing global expectations over time and the significance of new leadership approaches which are based on environmental sustainability has increased day by day. Green transformational leaders, one of the results of this evolution, are different from other leadership approaches in terms of their role in motivating employees and increasing their environmental awareness by integrating their environmental sensitivities into the organizational culture.

**Methods:**

This study, which aims to determine the mediating role of job satisfaction in the effect of green transformational leadership on intention to leave, was conducted with 391 people working in 4 and 5 star hotels in Antalya region. Data obtained by convenience sampling method was examined with Smart-PLS program. The study was shaped on the axis of structural equation modeling.

**Results:**

The findings showed that green transformational leadership has a negative effect on intention to leave and job satisfaction plays a mediating role in this relationship. In addition, green transformational leaders were found to increase employees’ job satisfaction, strengthen their commitment to the organization and reduce intention to leave.

**Discussion:**

The study shows that green transformational leadership has an effective structure not only in terms of green and environmental concepts but also in terms of organizational behavior. According to the Social Exchange Theory, green transformational leadership is thought to shape the job satisfaction of employees by interacting with them in accordance with moral and organizational norms.

## 1 Introduction

When the leadership literature is considered, it is realized that the focus of leadership approaches has shifted over time in parallel with the changing world. Many elements such as technology, law, culture, sociology and knowledge shape the concept of leadership; therefore, different definitions of leadership exist. Leadership is a conception that develops by being nourished by the environment in which people exist. As a result of people’s unconscious consumption and practices over the last 50 years, climate change has become clearly visible and affected the entire world (Rizvi and Garg, 2020). It is estimated that the damage caused by climate change will cost between 1.7 trillion and 3.1 trillion dollars by 2050. Studies indicate that the global cost of extreme weather events such as hurricanes, floods and heat waves in the last 20 years is estimated to be 2.8 trillion dollars. Moreover, when the situation of people who have to live in harsh conditions and die is taken into account, the situation becomes even worse (World Economic Forum, 2023). From this perspective, it is inevitable for a new leadership approach to develop. The pro-environmental behavior of green transformational leaders and the leaders’ influencing their employees to understand and learn the environmental protection values gradually are regarded as an adaptation process against climate change (Ding et al., 2023).

The requirement to implement a conscious green management system has now become a fundamental part of business strategies in different sectors and regions. At this point, the obligations imposed by protocols such as the United Nations Sustainable Development Goals, the Paris Climate Agreement and the European Union Green Deal have a great impact. It is realized that great efforts are shown to reduce greenhouse gases, which are especially dangerous for humanity and the environment (Çop et al., 2021). In recent years, as a result of natural disasters that occurred in various parts of the world due to climate change, millions of people had to leave their living spaces and thousands of people lost their lives (Sultana, 2023). In current world, rapid urbanization, increasing population, excess production-consumption, intense energy use and wasting natural resources have resulted in devastating damage to the nature; thus, sustainability has become more important than ever (Zeng et al., 2016). To ensure sustainability, green transformational leaders who create green product and process innovation, reduce the negative environmental impacts of organizations significantly and save money, time and resources (Singh et al., 2020) are needed. Carbon emission taxes practiced in many countries around the world seriously demonstrate the need for green transformational leadership.

Leadership is an important skill in terms of environmental management and sustainability in organizations (Le et al., 2024). In this regard, green transformational leadership differs from other types of leadership and makes it easier for the organization to adopt an environmentally friendly vision. In the same vein, it improves the environmental capacity in the organization to meet the expectations of all stakeholders (Abbas, 2024). For instance, a green transformative leader can use renewable energy sources actively to reduce the organization’s carbon footprint. Today, electricity generation with solar energy systems installed on the roof or in the garden of the organization’s work areas with high grant rates can be given as a good example of this (Saleem et al., 2024). While the electricity costs of the organization are reduced with the established system, a significant gain is achieved both inside and outside the organization by establishing better relations with the local government during the grant application, providing a more environmentally friendly image to the customers, increasing the profitability of the shareholders in terms of decreasing expenses and allowing employees to benefit from the profitability obtained (Al-Ghazali et al., 2022). Nevertheless, the distribution and impact of the gains achieved within the organization are not fully known.

It is observed that there is a gap in the literature in terms of determining the behavior of employees within the organization along with green transformational leadership practices (Rizvi and Garg, 2020; Singh et al., 2020; Çop et al., 2021; Du and Yan, 2022; Ozgul, 2022; Ding et al., 2023). Based on this gap, it is observed that green transformative leadership is often studied in terms of positive elements. Owing to the positive elements in the studies, particular attention is drawn to the increase in job satisfaction (Riva et al., 2021; Begum et al., 2022; Cui et al., 2023). Contrary to the literature in which people are regarded as machines and it is thought that they will fulfill every task given to them completely, the intention to leave variable is included as a variable in the present study. This stems from the fact that some failures may emerge in the process of creating job satisfaction. One of the concepts frequently studied together with green transformational leadership is green human resources management (Jia et al., 2018; Singh et al., 2020; Farrukh et al., 2022). The relationship between Green Human Resources Management and Green Transformational Leadership is related to how the vision and values created by leaders regarding environmental sustainability are reflected in the process of human resources management. While green transformational leaders increase environmental awareness in the organization, Green Human Resources Management ensures the integration of this awareness into daily HR practices. In the present study, with a different approach from the literature, the intention to leave the job was included in the model as an important and negative variable along with the positive behaviors in green human resources management. RBV theory (Barney, 2001) was utilized in order to explain the relationship between two variables. Specifically, RBV theory estimates that employees with high environmental sensitivity and more skills and knowledge will be a significant parameter for the organization to achieve sustainable superiority, and leaders are expected to make great efforts to keep these employees in the organization (Green et al., 2013). In this respect, it is thought that the two variables will act in an opposite manner to each other.

Social Exchange theory, which constitutes another theoretical source of the study, reveals that social behaviors in organizations emerge as a result of a process of change (Blau, 1964). Green transformational leaders are observed to show positive behavior towards their employees in return for their positive behavior towards their employees. While green transformational leader displays green behaviors according to social change theory, employees imitate these behaviors as leaders become role models for employees by sharing green values, communicating the importance of green practices clearly and applying ideas to address green-related problems (Lathabhavan and Kaur, 2023). Particularly in the relationship between employees and leaders, social exchange theory indicates that the employee’s positive work-related attitude depends on the organization’s trust on him, his approval and the leader. This situation suggests that the relationship between people in social life can be summarized as an exchange relationship (Chen and Yan, 2022). Briefly, based on the theory, meeting unwritten expectations such as fair treatment, fair compensation, promotion opportunities, safe working conditions, responsible business practices in return for good performance are realized to be an important variable in reducing employee turnover and ensuring job satisfaction (Bano et al., 2022).

When other studies are paid attention in the light of RBV and Social Exchange Theory, it is realized that another concept examined frequently along with green transformational leadership is green creativity (Chen and Chang, 2013; Mittal and Dhar, 2016; Li et al., 2020; Riva et al., 2021; Al-Ghazali et al., 2022; Ding et al., 2023). According to the literature, green creativity is regarded as an important prerequisite for achieving job satisfaction. Research findings suggest that green creativity enables employees to achieve high job satisfaction with high motivation resulting from the leader (Ding et al., 2023). When the relationship between green creativity and green transformational leadership is associated with the RBV theory (Barney, 2001), organizations developing valuable and rare resources to achieve environmental sustainability goals and using these resources to gain competitive advantage are considered to bear critical significance in terms of creating job satisfaction in employees (Riva et al., 2021). Likewise, within the scope of social exchange theory, it is realized that supporting the employee’s creativity by the leader will increase job satisfaction (Bano et al., 2022).

The present study, built on the connections between job satisfaction and intention to leave, aims to investigate the particular impact of green transformational leadership within the organization. This type of leadership emphasizes not only traditional transformational qualities but the environmental and sustainability concerns of organizations (Yukl and Gardner, 2020), as well. Bearing this in mind, the study offers the potential for a more comprehensive understanding of employee motivation and retention dynamics. In the study, 4 and 5 star hotels were selected as data collection points. Hotels were specifically chosen to collect samples because they accommodate people collectively and produce maximum waste in a limited area. Another reason for choosing four star and five star hotels is that they employ a high number of staff. The fact that hotels were frequently preferred in previous green transformational leadership studies is an element that encourages the study to be conducted in hotels (Mittal and Dhar, 2016; Moin et al., 2021; Riva et al., 2021; Çop et al., 2021; Ogretmenoglu et al., 2022; Sachdeva and Singh, 2023; Suliman et al., 2023; Xin and Wang, 2023; Aslam et al., 2024; Elshaer et al., 2024; Janjua et al., 2024; Nguyen et al., 2024). Due to the fact that Turkish tourism appeals more to Russian tourists, hotels in Turkey are generally more associated with sea tourism. For the hotels built by the sea, in the forest or in untouched areas with their unique beauty, limited access to municipal services such as sewage and garbage makes a sustainable hotel concept obligatory. As discharging sewage and garbage waste into the environment indiscriminately will not be welcomed by the customers there and will lead to various diseases, the practices are mandatory (Çop et al., 2021).The infrastructure of the created model is explained through Social Exchange Theory (Blau, 1964), Resource-Based View (RBV) Theory (Barney, 2001) and Hierarchy of Needs Theory (Maslow, 1943). Briefly, positive reflections of the policies implemented by the green transformation leader to achieve job satisfaction are encountered frequently in previous studies. However, this study is different from other studies in that it includes the intention to leave the job as a variable, which is a negative and undesirable behavior within the organization in order to achieve job satisfaction. This aspect of the study is expected to contribute to the literature on expanding the concept of green transformational leadership with organizational behavior issues.

## 2 Literature review

### 2.1 Green transformational leadership

The concept of sustainability, which is important for every living being, bears also great organizational significance. Organizations that are at the center of social change are important actors in terms of forming green awareness and spreading sustainability (Luan et al., 2022). In this respect, sustainability should be transformed into a basic and comprehensive concept that forms the basis for every organizational activity. This transformation is possible with the help of an environmentally conscious leadership approach. Leaders who can realize the necessary transformation for the spread of a sustainable and environmentally friendly (green) approach in every business function from supply to marketing become indispensable people in terms of organizational sustainability (Zhou et al., 2018).

Green leaders are defined in the literature as the ones who can create environmental awareness in the organization and among its members, are against the waste and adopt effectiveness and efficiency-oriented leadership approach. Furthermore, they are described as those who have environmental values, set an example for the members with their nature-friendly behaviors, shape organizational goals with environmental awareness and can turn this consciousness into a collective form (Ones and Dilchert, 2012). Transformational leaders can be defined as leaders who lead the change process in organizations where the current understanding must be abandoned in a structural sense and replaced with a new understanding. They are also regarded as those who can convince members for the necessity of change and ensure that the desired transformation becomes institutionalized and collective (Lin et al., 2022). Transformational leaders ensure that the organization’s values, goals and objectives are understood and assimilated by members through intellectual influence, individualized interest, charisma and inspiration in their leadership processes (Ozgul, 2022).

Green transformational leaders can be defined as a combination of these two leadership approaches which are described. In this regard, green transformational leaders can be described as leaders who manifest themselves in organizational structures where the necessity of transformation is at the point of environmental understanding and who place their environmental awareness at the center of organizational transformation (Suliman et al., 2023). Green transformational leadership refers to a leadership approach which raises awareness of members towards environmental sustainability requirements. Besides, they are expected to motivate those members to behave in accordance with these requirements, influence members to engage in environmentally friendly behavior with their actions and inspire them in line with the desired result (Chen and Chang, 2013). According to another definition, green transformational leadership is a type of leadership that can persuade employees to achieve organizational goals in the context of environmentalism, stimulate green consciousness in employees, convert that consciousness into behavior and transform those behaviors into a sustainable form (Kerse et al., 2021).

Apart from carrying out environmentally conscious organizational transformation, green transformational leaders make significant contributions to the sustainable development of the environment in which they operate. As is known, the continuity of an organization’s existence is possible with the continuity of its resources (Du and Yan, 2022). Especially in present world, where competitive severity is extremely intense, organizations can maintain their existence and provide sustainable competition with their effective and efficient activities. Green transformational leadership assumes an important function at this point and creates space for businesses to achieve low consumption, low cost and sustainable competition through avoidance of waste and conscious use of resources (Norton et al., 2017).

Green transformational leaders are the ones who mediate employees in terms of feeling sensitive to environmental issues in their organizational activities as well as their individual tendencies. They also transform their sensitivity on environmental issues into the necessity of a collective vision by carrying beyond reward-punishment practices and are able to set an example for their members and integrate with them in terms of displaying environmentally friendly behaviors (Jia et al., 2018).

Moreover, when studies on green transformative leadership are paid attention, these practices are also realized to have effects that contribute to organizational sustainability such as triggering collective consciousness in employees (Kura, 2016), increasing the level of subjective well-being (Biswas et al., 2021), increasing job satisfaction (Li et al., 2020) and providing organizational belonging (Norton et al., 2015) in addition to their environmental awareness and sensitivity effects.

### 2.2 Intention to leave the job

The intention to leave the job is a frequently encountered phenomenon in organizations where job satisfaction cannot be achieved. As a matter of fact, that intention arises as a result of employees’ dissatisfaction with working conditions (Felicia et al., 2023). The intention to leave the job refers to individuals’ conscious and determined state of leaving the organization. According to Social Exchange Theory, employees have a positive expectation in return for the work they do and failure to meet this expectation constitutes the focal reason for the intention to leave the job (Sypniewska et al., 2023). At this point, it is possible to explain the intention to leave the job in three different ways such as economic reasons, intra-organizational reasons and individual reasons. Economic reasons depend on variables such as the prosperity of the country, the possibility of finding a job again and the risk of an economic crisis. Reasons within the organization can be stated as working hours, transportation facilities, inequality in the promotion and reward system and poor management practices. Individual reasons are explained by many factors such as not being able to receive the expected wage, family relationships, age, education and health problems (Chen et al., 2021). According to Social Exchange Theory, a decrease in the intention to leave the job is expected with the positive effect of green transformational leaders on employees.

The rates of intention to leave are observed to be approximately twice as high in the service sector as other sectors (Ghani et al., 2022). Employees are resources that are difficult to find and obtain because they constitute the main capital structure of businesses. Especially in the service sector, the existence of a close relationship between the quality of work outputs and employees makes employees a critical point for businesses operating in this sector (Seriki, 2023). The present study was inspired by the Resource-Based View (RBV) due to the fact that employees are one of the strategic resources that are valuable for the organization in terms of competitive advantage and performance, and are rare and difficult to imitate by competitors in the market. Within the scope of RBV, job satisfaction and leadership are regarded as critical resources in environmental management in the organization and are thought to be effective tools in reducing the intention to leave (Singh et al., 2020). In accordance with the literature, green transformational leaders are expected to exhibit great efforts to reduce employees’ intention to leave the job by paying special attention to human resources management (Jia et al., 2018; Singh et al., 2020; Farrukh et al., 2022). Leaders strive to retain human resources, which is one of the rare resources, due to the fact that employee turnover creates a burden on other employees and has psychological effects as well as working harder (Nguyen et al., 2024). The previous studies also confirm that, the intention of employees to leave the job will increase if leaders do not reduce their negative psychological perceptions. Li et al. (2021) conducted a study with 531 employees in the energy sector and found a negative and significant effect between green transformational leadership and the intention to leave. In the study conducted by Torun (2022) with 164 hospital employees, a negative and significant effect was detected between transformational leadership and intention to leave. On the other hand, in another study carried out by Gom et al. (2021) with 162 hotel employees, it was observed that there was a negative and significant relationship between transformational leadership and intention to leave. Due to the characteristics of transformational leadership, it is expected that the intention to leave will decrease as a natural reflection of supporting and inspiring employees and trying to reduce the effects of a stressful work environment (Çop et al., 2021). When the literature is examined, similar results are obtained in many studies, which shows that organizations provide psychological and financial support to employees, who are rare resources, to prevent them from exhibiting the intention to leave their job (Green et al., 2013). Inspired by RBV, H_1_ hypothesis was formed in accordance with the efforts that leaders are expected to make so as to retain employees, who are the most basic rare resource of the organization.

**H**_**1**_. Green Transformational Leadership has significant effect on the Intention to Leave the Job.

### 2.3 The mediating role of job satisfaction

Job satisfaction, which is based on Maslow’s hierarchy of needs theory (Sypniewska, 2014), is one of the topics studied the most in the field of organizational behavior (Hofmans et al., 2013). Due to its multidimensional structure, job satisfaction has been a concept whose definitions have been handled with different approaches (Wanous and Lawler, 1972). Job satisfaction is defined by Vroom (1964) as emotional orientations of individuals regarding their roles at work. Meanwhile, while George and Jones (2014) describe job satisfaction as the sum of people’s feelings and beliefs about their current jobs, Brief (1998) defined it as an internal state that the employee expresses by evaluating a job positively or negatively when he/she looks from an emotional and/or cognitive perspective.

Job satisfaction, defined as an employee’s overall evaluation of his work experiences and perceptions (Locke, 1978), is an important psychological state. It has profound effects on organizational success. Job satisfaction includes both positive and negative attitudes towards one’s job and influences employee commitment, performance and behavior considerably in the organizational context (Al-Asadi et al., 2019). Job satisfaction provides internal positive emotions and individual contributions to the employee (Lan et al., 2019); It enables people to have healthy communication outside of work, have high social adaptation (Smith et al., 1969) and have a satisfying life (Hackman and Oldham, 1980; Judge et al., 1998). Moreover, it has been revealed in many studies that satisfied employees tend to be more productive and creative in the working environment and are less likely to leave their jobs (San Park and Hyun Kim, 2009; Lee, 2020) and has been stated that job dissatisfaction is one of the antecedents of the intention to leave the job (Tett and Meyer, 1993; San Park and Hyun Kim, 2009). In this respect, it has been stated that a satisfied employee will be happy individually, have high organizational commitment and display behaviors with a low tendency to leave the job and for absenteeism (Rai and Maheshwari, 2020).

Job satisfaction is a multidimensional structure that can be measured in various ways and is closely related to two key concepts particularly discussed in this study including leadership style (Bogler, 2001; Mohammad Mosadegh Rad and Hossein Yarmohammadian, 2006; Li et al., 2019) and intention to leave the job (Judge and Piccolo, 2004; Podolsky and Hackett, 2021). In particular, transformational leadership has a positive impact on job satisfaction of employees (Labrague et al., 2020; Moin et al., 2021). It is frequently emphasized in the literature that this leadership style, which stands out with its inspiring, supportive, motivating and visionary characteristics of its followers, has a positive effect on job satisfaction (Bass, 1985; Avolio et al., 1999). The study carried out by Ansari and Khan (2024) with 418 employees in the pharmaceutical industry in Pakistan demonstrated that green transformational leadership has a significant and positive effect on job satisfaction, which is supported by the RBV theory. In the study conducted by Aburumman and Alrweis (2024) on 314 people working in a 5-star hotel in Jordan, it was found that transformational leadership had a significant and positive effect on job satisfaction, and that job satisfaction had a mediating role in the effect of transformational leadership on job performance. Belias et al. (2022) carried out a study on 209 employees in 3- and 4-star hotels in Greece and found that transformational leadership had a significant and positive effect on job satisfaction. Moin et al. (2021), in his study with 534 hotel employees in Malaysia, observed that green transformational leadership had a moderate, significant and positive effect on job satisfaction, which was explained through Social Exchange Theory. It is possible to multiply the examples, and in accordance with the literature review, it is necessary to explain where the leader’s effectiveness in job satisfaction comes from. By investing in individual development and encouraging a sense of purpose, transformational leaders increase employees’ appreciation of their work and in this way pave the way for higher job satisfaction (Gessesse and Premanandam, 2023), which strengthens organizational stability by increasing organizational commitment.

Green transformational leadership, as stated before, is characterized by striving to spread the green understanding throughout the organization by stimulating employees to learn new skills and technologies. The green transformational leader also persuades employees to place the green goals of the organization ahead of their individual goals. Briefly, green transformational leaders are creative and innovative leaders who provide job satisfaction by combining the goals of employees and the goals of the organization thanks to their visionary features (Ding et al., 2023). The assumption that parties enter into and maintain social relationships with the expectation of being rewarded (i.e., respect, honor, friendship, consideration etc.) forms the basis for Social Exchange Theory (Blau, 1964). Inspired by Social Exchange Theory, H_2_ hypothesis was formed because the green transformative leader is expected to motivate and influence employees positively with his visionary approach.

**H**_**2**_. Green Transformational Leadership has a significant effect on Job Satisfaction.

According to the Social Exchange Theory, intention to leave the job indicates a negative situation for the organization that occurs when employees’ expectations are not satisfied. According to theory, the intention to leave the job is due to the belief that employees will not be rewarded for their efforts (Sypniewska et al., 2023). Hennicks et al. (2024) conducted a study with 403 employees in a public institution in South Africa and found that job satisfaction reduced their intention to leave the job. In the study conducted by Tutan and Kökalan (2024) on healthcare workers in Turkey, it was found that there was a negative, significant and high level relationship between job satisfaction and intention to leave. Zientara et al. (2024) carried out a study with 263 hotel employees in the United Kingdom and suggested that job satisfaction had a high, negative and significant effect on intention to leave. In the study conducted by Davras and Davras (2024) with 432 hotel employees in Turkey, job satisfaction was observed to have a high, negative and significant effect on intention to leave. Studies conducted in Turkey show that situations such as late payment of salaries, as well as difficulties in paying overtime and fringe benefits, are common in the sector (Evcimen et al., 2022). Although there are many judicial decisions and legal regulations on this issue, there is no serious change in the situation (Cil, 2007). Considering that Turkey has been one of the five countries with the highest inflation in the world in recent years, it is expected that the intention to leave the job due to economic reasons will increase. When the stated reasons for Turkey and the Social Exchange Theory are examined, it is expected that ensuring job satisfaction will reduce the intention to leave the job. In this respect, H_3_ hypothesis was formed in accordance with the specified literature.

**H**_**3**_. Job Satisfaction has a significant effect on the Intention to Leave the Job.

In many recent studies, it is realized that green transformational leadership is frequently studied with concepts such as green innovation and green human resources. The numerical scarcity of studies evaluating green transformational leadership in terms of organizational behavior is noteworthy (Çop et al., 2021). Previous studies have found that job satisfaction plays a partial mediating role in the relationship between leadership behavior and the intention to leave the job (Promchart and Potipiroon, 2020), which highlights the critical function of leadership behavior in shaping employees’ job satisfaction and therefore influencing organizational outcomes. According to various studies, transformational leadership has a positive impact on job satisfaction, which leads to a decrease in the intention of employees to leave the job (Berson and Linton, 2005; Nemanich and Keller, 2007; Manoppo, 2020; Kaymakcı et al., 2022). This indirect relationship indicates that strong leadership characteristics lead to high job satisfaction and low intention to leave the job (Roche et al., 2015; Worthy et al., 2020). In the present study, the effects of green transformational leadership on organizational behavior are examined in the light of Social Exchange Theory, RBV Theory and Motivation theories. In this regard, H4 hypothesis is formed and an attempt is made to measure the effect of green transformational leadership on the intention to leave the job and the mediating effect of job satisfaction.

**H**_**4**_. Job Satisfaction has a mediating role in the effect of Green Transformational Leadership on the Intention to Leave the Job.

**Figure 1:**
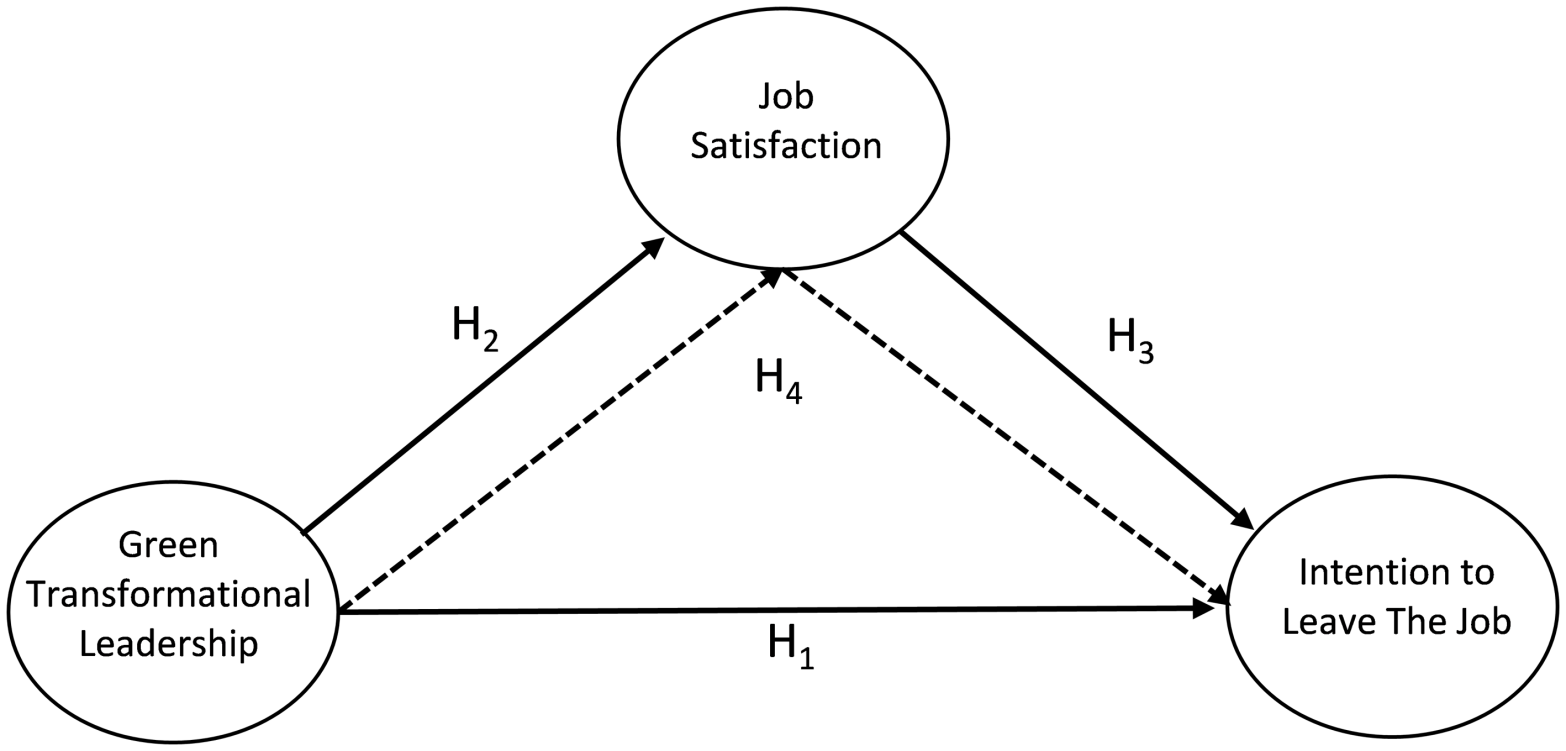
Research model.

The model that emerged as a result of forming the hypotheses is presented in Figure 1.

## 3 Materials and methods

The study was conducted with employees in hotels with four and five stars in Antalya region and was designed to see green transformative leadership from the employees’ perspective without including the opinions of the management staff. In the study, four star and five star hotels were preferred as data collection areas owing to their high accommodation capacities and comprehensive business units. Furthermore, creating a lot of waste in a limited area is one of the reasons why four and five star hotels are preferred for the study and it is realized that this is evaluated as an important factor in many studies with the theme of environment (Mittal and Dhar, 2016; Moin et al., 2021; Riva et al., 2021; Çop et al., 2021; Ogretmenoglu et al., 2022; Sachdeva and Singh, 2023; Suliman et al., 2023; Xin and Wang, 2023; Aslam et al., 2024; Elshaer et al., 2024; Janjua et al., 2024; Nguyen et al., 2024). The reason for this planning was to determine green transformative leadership in the eyes of employees. Permission for the study was received from Osmaniye Korkut Ata University Social Sciences Ethics Committee on 02 August 2023. In order to collect data, 41 hotels in Antalya region were first determined, and with the feedback from the human resources managers of 7 four-star and 11 five-star hotels, the study was carried out in 18 hotels in total. The number of people employed in the hotels in question varies between 81 and 1.230 people, and there are over 10.000 employees in total. After getting the necessary permissions from the hotel managers, the survey form of the study was shared digitally with the human resources managers and delivered to the participants through the human resources managers using the method of convenience sampling. The survey was carried out between September 2, 2023 and March 12, 2024 and a total of 459 people were reached. The data obtained from 68 people having managerial positions were not included in the study and the study conducted with the remaining 391 participants. The number of data obtained is adequate to represent the whole in a statistical sense in accordance with the Cochran (1977) formula. The data obtained from a sufficient number of participants were analyzed with the Smart PLS program.

Smart PLS is a Partial Least Squares Structural Equation Modeling (PLS-SEM) program. Since its introduction for business research by Chin (1995) in the mid-1990s, PLS-SEM has increasingly taken its place in the literature (Khan et al., 2020). PLS-SEM is an important method preferred in social sciences owing to its ability to analyze cause-effect relationships between latent variables, complex and problematic models, and take into account various forms of measurement error simultaneously (Hair et al., 2017a). SEM is an accepted paradigm which is used to measure the validity of theories with empirical data (Hoque and Sorwar, 2017). The previous adoption of the same method in other studies on green transformative leadership (Aslam et al., 2024; Awan et al., 2023; Bhatti et al., 2020; Farrukh et al., 2022; Kusi et al., 2021; Niazi et al., 2023; Tian et al., 2023) encouraged the use of PLS-SEM in the present study. This is because it is thought that using the same method with other studies will facilitate comparison.

For testing the model, scales previously translated into Turkish by other researchers were preferred. In this respect, for measuring green transformational leadership, the scale developed by Chen and Chang (2013) and translated into Turkish by Kerse et al. (2021) was used. The reliability and validity of the scale developed by Chen and Chang (2013) are considered to be high with a value of 0.90 according to Cronbach’s Alpha. There are a total of six questions in the scale with statements such as “The leader of the green product development project inspires the project members with the environmental plans.” The scale is one-dimensional and was developed in a 5-point Likert form.

The scale developed by Rosin and Korabik (1991) and translated into Turkish by Gercek et al. (2015) was utilized in order to measure the intention to leave the job. The reliability and validity of the scale developed by Rosin and Korabik (1991) is considered to be moderate with a value of 0.82 according to Cronbach’s Alpha. In the scale, for example, there exist a total of four questions with expressions such as “At this time in your career, would you want to quit this job if it were possible?.” The scale is one-dimensional and was developed in a 5-point Likert form.

The scale developed by Judge et al. (1998) and translated into Turkish by Başol and Comlekci (2020) was utilized in order to measure the job satisfaction. The reliability and validity of the scale developed by Judge et al. (1998) is considered to be moderate with a value of 0.88 according to Cronbach’s Alpha. In the scale, for example, there exist a total of five questions with expressions such as “‘I feel fairly well satisfied with my present job.” The scale is one-dimensional and was developed in a 5-point Likert form.

## 4 Results

It was observed that the majority of the sample with which the survey was performed was male (60.60%), the number of married people (71.90%) was higher than the single ones and the age of the participants was mostly between 18 and 40 (83.70%). Details of demographic information are illustrated in Table 1.

**Table 1:** Demographic information.

Demographic	Variable	*n*	%
Gender	Female	154	39.40
	Male	237	60.60
Marital Status	Married	281	71.90
	Single	110	28.10
Age	Between 18 and 30 Ages	179	45.80
	Between 31 and 40 Ages	148	37.90
	Between 41 and 50 Ages	52	13.30
	51 Ages and Above	12	3.00
Level of Education	High School and Below	154	39.40
	Undergraduate	131	33.50
	Graduate	97	24.80
	Postgraduate	9	2.30
Sectoral Experience	5 years and Below	132	33.80
	Between 6 and 10 years	159	40.70
	Between 11 and 15 Years	54	13.80
	Between 16 and 20 Years	36	9.20
	21 Years and Above	10	2.50
Department	Kitchen	42	10.70
	Reception	24	6.10
	Restaurant Service	107	27.40
	Housekeeping	98	25.10
	Technical	27	6.90
	Other	93	23.80
Income	Between 15.000 and 20.000 Turkish Liras	218	55.80
	Between 20.001 and 30.000 Turkish Liras	112	28.60
	Between 30.001and 40.000 Turkish Liras	33	8.40
	Between 40.001 and 50.000 Turkish Liras	18	4.60
	50.001 Turkish Liras and Above	10	2.60

Cronbach’s Alpha, Composite Reliability (CR) and Average Variance Extracted (AVE) values were used so as to measure the reliability, validity and consistency of the scales performed with the sample. Cronbach’s Alpha, Composite Reliability (CR) and rho_A values being over 0.70 indicate the appropriateness of reliability, validity and consistency. The fact that the AVE value is above 0.50 and that it is lower than the Composite Reliability (CR) and rho_A values also shows construct validity (Sarstedt et al., 2014). Detailed information about these values is displayed in Table 2.

**Table 2:** Factor load values, validity and reliability.

Item	Factor loading	Median	Standard deviation	Kurtosis	Skewness
Green Transfromational Leadership Scale					
Cronbach’s Alpha = 0.967, rho_A = 0.969, CR = 0.973, AVE = 0.859					
GTL1	0.899	3.033	1.180	−0.998	−0.177
GTL2	0.933	3.028	1.176	−1.004	−0.225
GTL3	0.953	3.097	1.147	−0.911	−0.252
GTL4	0.947	3.161	1.187	−0.905	−0.333
GTL5	0.905	3.130	1.127	−0.843	−0.302
GTL6	0.923	3.133	1.161	−0.878	−0.281
Job Satisfaction Scale					
Cronbach’s Alpha = 0.924, rho_A = 0.935, CR = 0.943, AVE = 0.768					
Satisfaction1	0.897	3.660	1,065	0.123	−0.792
Satisfaction2	0.918	3.483	1.108	−0.298	−0.553
Satisfaction3	0.825	3.944	1.012	1.016	−1.104
Satisfaction4	0.892	3.547	1.076	−0.231	−0.630
Satisfaction5	0.847	3.082	1.148	−0.759	−0.130
The Intention to Leave the Job					
Cronbach’s Alpha = 0.838, rho_A = 0.873, CR = 0.893, AVE = 0.680					
Leave1	0.927	3.043	1.338	−1.220	0.055
Leave2	0.906	3.018	1.339	−1.271	0.038
Leave3	0.748	2.650	1.316	−0.956	0.443
Leave4	0.693	2.629	1.289	−0.443	0.781

It is significant that the factor load values of the items in the scales are above 0.70 in terms of ensuring validity and reliability. As a result of the analysis, it is realized that the scales have the necessary values for analysis. However, discriminant validity, indicating that there is no combination between the scales, should also be calculated (Hair et al., 2017). The Fornell-Larcker criterion for variance-based structural equation modelling such as partial least squares and examination of cross-loadings is the dominant approach for the assessment of discriminant validity. In addition, Heterotrait-Monotrait Ratio (HTMT) is regarded as the most preferred method. HTMT being below 0.90 indicates discriminant validity (Hair et al., 2019). Values for discriminant validity are shown in Table 3.

**Table 3:** Discriminant validity.

	Fornell-Larcker criterion			Heterotrait-Monotrait Ratio (HTMT)		
	1	2	3	1	2	3
Green Transformational Leadership	0.927					
Job Satisfaction	0.416	0.876		0.435		
The Intention to Leave the Job	−0.358	−0.628	0.825	0.391	0.695	

As the convenience of the scales was at the desired standards, the convenience of the model was tested. The Standardized Root Mean Square (SRMR) value being below 0.80 and the Normed Fit Index (NFI) value being above 0.80 indicate the goodness of fit of the model (Byrne, 2016). Values regarding the model are presented in Table 4.

**Table 4:** Model goodness of fit values.

Items	Saturated model	Estimated model
SRMR	0.053	0.053
d_ULS	0.335	0.335
d_G	0.228	0.228
Chi-Square	521.487	521.487
NFI	0.911	0.911

Q2 test was performed in order to determine the quality of the analysis and the value obtained is different from zero (0 < Q2), which reveals the significance of the test (Hair et al., 2017). Values for Q2 test are shown in Table 5.

**Table 5:** Q^2^ test result.

Latent variable	R^2^	R^2^ Adj.	Q^2^
Job Satisfaction	0.173	0.171	0.129
The Intention to Leave the Job	0.406	0.403	0.268

**Figure 2:**
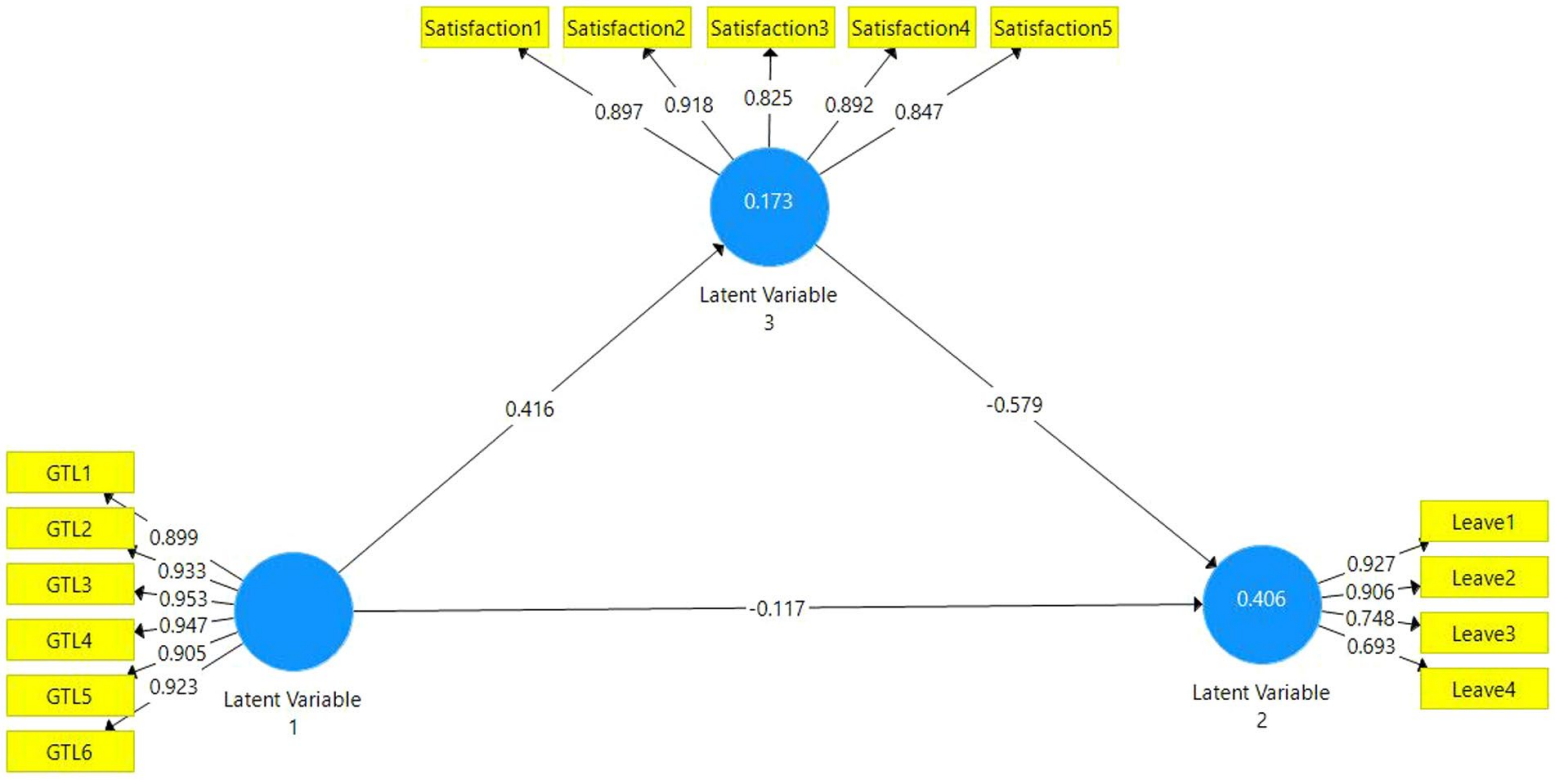
Path diagram.

Hypothesis tests were performed by using the bootstrapping method in accordance with the literature and by selecting a sample size of 5,000 as structural equation modelling. Despite the fact that there are different applications other than this method such as the Baron and Kenny and Sobel test, this method was preferred due to the fact that it is easy to use (Sagbas et al., 2023; Korkmaz and Altintas, 2024). The visual of the analysis is presented in Figure 2.

The detailed information regarding hypothesis analyses are presented in Table 6.

**Table 6:** Hypothesis test result.

Path analysis	Estimate	Standard deviation	*t*-values	*p*	Support
Green Transformational Leadership → Intention to Leave	−0.117	0.050	2.350	**0.019**	**H**_**1**_ **Accepted**
Green Transformational Leadership → Job Satisfaction	0.416	0.048	8.650	**0.000**	**H**_**2**_ **Accepted**
Job Satisfaction → Intention to Leave	−0.579	0.043	13.578	**0.000**	**H**_**3**_ **Accepted**
Green Transformational Leadership → Job Satisfaction → Intention to Leave	−0.241	0.035	6.958	**0.000**	**H**_**4**_ **Accepted**

Bold values indicate that the hypotheses are accepted with a *p* value below 0.05.

A negative significant effect of green transformational leadership on intention to leave the job was determined, and therefore, H1 hypothesis was accepted, which is possible to explain with social exchange theory. According to that theory, employees are expected to follow the green transformative leader’s behavior and thoughts, which in turn increases employees’ trust and expectations (Şahin and Demiral, 2023). It is an expected behavior that the intention to leave the job will decrease as a result of the positive behavior of the leader. It is realized that similar results were obtained in previous studies (Sahu et al., 2017; Alorhiri et al., 2019).

The positive significant effect of green transformational leadership on job satisfaction was observed and the H2 hypothesis was accepted. In other words, leader support increases job satisfaction of employees, which is possible to explain through RBV theory. Transformational leaders are generally those who transform values, desires and priorities of their employees and motivate them to perform above expectations. For this reason, it is observed that this leadership approach leads to a high job satisfaction impact on employees, which provides the organization with a unique and inimitable competitive advantage (Choi et al., 2011).

A negative significant effect of job satisfaction on intention to leave was observed; hence the H3 hypothesis was accepted. In this regard, according to the hierarchy of needs theory, employees who experience job satisfaction are realized to make efforts in order to improve their current position at work as opposed to their intention to leave the job. The result obtained is found to be compatible with the literature in this respect (Amunkete and Rothmann, 2015).

The mediating effect of Job Satisfaction on the negative significant effect of Green Transformational Leadership on Intention to Leave the Job was realized; thus the H4 hypothesis was accepted. It is possible to explain the result of the model formed by three different variables according to social exchange theory, RBV theory and hierarchy of needs theory. The result obtained bears importance in terms of explaining the reflections of green transformative leadership within the organization.

## 5 Discussion

The present study, which is based on the connections between job satisfaction and intention to leave the job, aims to investigate the particular effect of green transformational leadership within the organization. In this respect, the study conducted with hotel employees working at hotels with 4 and 5 stars will make contribution to the literature. It is thought that the significance of the green transformational leadership is increasing day by day especially in the context of climate change and has become a requirement for organizations. In accordance with the Paris Climate Agreement, zero carbon practices constitute the legal dimension of the obligation (Çop et al., 2021; Ding et al., 2023). The incentives for green production that have started to be practiced by states and the penalties imposed against carbon emissions will enable the importance of green transformative leadership to become more evident and come to the fore in the future. The results obtained through the study are given below.

With the first hypothesis, the negative effect of green transformational leadership on the intention to leave the job was observed. When the characteristics of the green transformational leader are considered, it is thought that leaders’ guiding and rewarding employees in a moral and normative relationship will help to reduce the intention to leave the job within the scope of social exchange theory (Sahu et al., 2017; Alorhiri et al., 2019). Based on the RBV theory, the intention to leave the job is expected to be reduced within the organization when leaders protect the human resources they have and transform the organization in a way as to exhibit green behavior (Jia et al., 2018; Singh et al., 2020; Farrukh et al., 2022). In the light of this expectation, the study proves that the leader strives for the development of the organization by protecting its human resources.

Through the second hypothesis, the positive effect of green transformational leadership on job satisfaction was observed. It is possible to regard green transformational leadership as a positive type of leadership. Therefore, employees are expected to be rewarded and appreciated as a result of the social exchange between the leader and the employee (Chen and Chang, 2013; Kerse et al., 2021; Ding et al., 2023). Moreover, it can be put forward that the situation in question is nourished by Maslow’s Hierarchy of Needs Theory in that employees’ needs for self-actualization and appreciation at work enable this inference (Maslow, 1943). The result obtained is observed to be consistent with the literature as it is associated with job satisfaction of employees who are appreciated and rewarded by the leader (Choi et al., 2011).

The third hypothesis determined the negative effect of job satisfaction on intention to leave. When the literature is considered, it is not a very common situation for an employee who is satisfied to leave the job. Due to the fact that two concepts are often used in opposition to one other, the result obtained reveals that human resources are a quite important element that leads to a competitive advantage, thus decreasing employees’ intention to leave the job and increasing job satisfaction (Singh et al., 2020). The result obtained indicates that it is necessary for the organization to retain its existing human resources, which are rare and provide a competitive advantage, in accordance with the resource-based view theory.

With the fourth hypothesis, the mediating effect of job satisfaction on the effect of green transformational leadership on the intention to leave the job was found. With the model formed, it is realized that 24.1% of the negative effect of green transformational leadership on intention to leave could be explained through job satisfaction. The result obtained suggests in accordance with the social exchange theory that with the support of the green transformation leader, job satisfaction increases and in parallel, the intention to leave the job decreases.

The research findings are remarkable from theoretical and practical perspective they put forward in terms of both employees and organizations. In this respect, in terms of theoretical results, the study has revealed the effect of green transformational leadership on employee behavior in line with social exchange theory, resource-based view and hierarchy of needs theories by addressing the relationships between the concepts of green transformational leadership, job satisfaction and intention to leave the job. For this reason, green transformational leaders are observed to increase employees’ job satisfaction, which in turn reduces intention to leave the job. This has shown that leaders strengthen employees’ commitment to their working environment and reinforce employees’ desire to stay in the organization. Moreover, the study has made another important contribution to the literature by providing a new understanding of the connection between green transformational leadership and organizational behavior as it has revealed that this type of leadership not only encourages environmentally friendly behavior of employees, but also contributes to the long-term sustainability of organizational success.

From a practical perspective, on the other hand, the study suggests very important findings, especially for organizations operating in the service sector such as hotel businesses. In this regard, organizations that adopt green transformational leadership practices can increase employee job satisfaction and reduce the rates of intention to leave the job. This situation can stand out as a critical strategy for organizations both in terms of ensuring employee continuity and reducing the costs. Additionally, leaders setting a model for implementing environmentally friendly policies can increase employees’ support for these policies. This support has the potential to not only encourage environmentally responsible organizational behavior, but to increase employee motivation and productivity, as well.

In this respect, green transformative leadership stands out as an important approach especially for organizations which want to carry out environmentally friendly projects and practices. As for the applicable strategies, it can be recommended that leaders receive environmental awareness training and lead their employees on these issues.

## 6 Conclusion

When the literature is paid attention, it is found that green transformational leadership is studied frequently with variables such as green innovation and environmental performance. In this regard, the study can be claimed to encourage a small step towards expanding the concept of green transformational leadership, which proceeds in a shallow framework, with the help of organizational behavior issues. Within the scope of green transformational leadership, the study was carried out as it was observed that there was less tendency to focus on organizational behavior issues while the concepts of green human resources (Jia et al., 2018; Singh et al., 2020; Farrukh et al., 2022), green innovation (Begum et al., 2022; Luan et al., 2022; Cui et al., 2023; Hanif et al., 2023; Pham and Pham, 2023; Sánchez-García et al., 2023) and green creativity (Chen and Chang, 2013; Mittal and Dhar, 2016; Riva et al., 2021; Al-Ghazali et al., 2022; Ding et al., 2023) were studied frequently. In this respect, the study is expected to make contribution to the literature.

The study is different from other studies in the literature in that it associates green transformational leadership with various variables related to green and the environment. In the literature review, it is realized that whether the green transformational leadership approach affects the job satisfaction of employees and its role in employee continuity for the health of the organization has not been studied before. Whether a research has been conducted before or not is not actually an important issue. The importance of a study can be measured in terms of its theoretical and scientific contribution to the literature. In this regard, the present study indicates that green transformational leadership has an effective structure not only in terms of green and environmental concepts but also in terms of organizational behavior. Green transformational leadership is thought to shape employees’ job satisfaction by interacting with employees in accordance with moral and organizational norms according to Social Exchange Theory. Meanwhile, it is found that this finding has an impact on retaining rare resources (i.e., employees) and providing competitive advantage when associated with RBV.

On the other hand, this study also includes some limitations. First of all, the fact that the study was conducted cross-sectionally because of its cost-effectiveness does not make it possible to make causal inferences and to obtain results that may change over time. For this reason, future studies could be carried out longitudinally and examine dynamic changes in the relationships between variables. It may be a matter of criticism that the study was conducted at hotels within the service sector. However, hotels are businesses that have more negative effects on nature owing to the fact that they host thousands of people at the same time and are generally located in nature (Mittal and Dhar, 2016). Bearing this in mind, hotels that are in touch with nature by hosting sea tourism were preferred in this study and further studies are recommended to be carried out by other researchers on points such as industrial facilities and public institutions. Another suggestion for further studies could be to study the issues of rumination, sustainability, digital transformation and corporate social responsibility of the concept of green transformative leadership.

## Data Availability

The raw data supporting the conclusions of this article will be made available by the authors, without undue reservation.
